# The effect of UV-B on Arabidopsis leaves depends on light conditions after treatment

**DOI:** 10.1186/s12870-015-0667-2

**Published:** 2015-11-25

**Authors:** Olga Sztatelman, Joanna Grzyb, Halina Gabryś, Agnieszka Katarzyna Banaś

**Affiliations:** Department of Plant Biotechnology, Faculty of Biochemistry, Biophysics and Biotechnology, Jagiellonian University, Gronostajowa 7, Krakow, 30-387 Poland; Current address: Institute of Biochemistry and Biophysics, Polish Academy of Sciences, Warszawa, 02-106 Poland; Laboratory of Biological Physics, Institute of Physics, Polish Academy of Sciences, Al. Lotników 32/46, Warszawa, 02-668 Poland; The Malopolska Centre of Biotechnology, Jagiellonian University, Gronostajowa 7, Krakow, 30-387 Poland

**Keywords:** Cell death, Chlorophyll degradation, Light, Photosynthesis, Senescence, UV-B

## Abstract

**Background:**

Ultraviolet B (UV-B) irradiation can influence many cellular processes. Irradiation with high UV-B doses causes chlorophyll degradation, a decrease in the expression of genes associated with photosynthesis and its subsequent inhibition. On the other hand, sublethal doses of UV-B are used in post-harvest technology to prevent yellowing in storage. To address this inconsistency the effect of short, high-dose UV-B irradiation on detached *Arabidopsis thaliana* leaves was examined.

**Results:**

Two different experimental models were used. After short treatment with a high dose of UV-B the *Arabidopsis* leaves were either put into darkness or exposed to constant light for up to 4 days. UV-B inhibited dark-induced chlorophyll degradation in *Arabidopsis* leaves in a dose-dependent manner. The expression of photosynthesis-related genes, chlorophyll content and photosynthetic efficiency were higher in UV-B -treated leaves left in darkness. UV-B treatment followed by constant light caused leaf yellowing and induced the expression of senescence-related genes. Irrespective of light treatment a high UV-B dose led to clearly visible cell death 3 days after irradiation.

**Conclusions:**

High doses of UV-B have opposing effects on leaves depending on their light status after UV treatment. In darkened leaves short UV-B treatment delays the appearance of senescence symptoms. When followed by light treatment, the same doses of UV-B result in chlorophyll degradation. This restricts the potential usability of UV treatment in postharvest technology to crops which are stored in darkness.

**Electronic supplementary material:**

The online version of this article (doi:10.1186/s12870-015-0667-2) contains supplementary material, which is available to authorized users.

## Background

Beside visible light the solar radiation which strikes the Earth’s atmosphere also contains ultraviolet (UV) and infrared irradiation. Based on the biological effects it induces, UV is divided into UV-C (100–280 nm), UV-B (280–320 nm) and UV-A (320–400 nm). UV-C, the most dangerous, is completely absorbed by the ozone layer in the atmosphere. As a consequence, UV-B is the shortest wavelength component of the sunlight which reaches the surface of the Earth. As an integral part of solar radiation, UV always accompanies visible light. This is of special importance for plants which are both sessile and photosynthetic organisms. The UV-B range is absorbed by many constituents of the cell with harmful consequences. UV-B is cytotoxic, damaging the cell at many levels, including nucleic acids, lipids, photosynthetic pigments and proteins [1]. Higher levels of UV-B cause the production of reactive oxygen species (ROS) and activate general stress signaling pathways [2]. Moreover, the UV-B-dependent formation of dimers between adjacent pyrimidines in DNA strands may be both mutagenic and genotoxic due to blocking the progress of DNA polymerase. As a result, the exposure of plants to high levels of UV can lead to cell death dependent on ROS signaling (for a review see: [3]).

The depletion of the ozone layer has resulted in an increase in the level of UV-B reaching the Earth’s surface. That is why the impact of this wavelength range on living organisms started to be intensively investigated in the eighties. Most experiments have been performed in growth chambers with relatively low photosynthetically active radiation (PAR) supplemented with a high dose of UV-B [4]. They showed a very strong impact of UV-B on the content of photosynthesis dependent pigments, the activity of photosynthetic enzymes and photosynthetic efficiency (for a review see: [5]). UV-B affects Photosystem II (PSII) to much greater extent than Photosystem I [1]. The degradation of integral components of PSII reaction centers, including D1 and D2 proteins, is an extensively studied aspect of the effect of UV-B on photosynthesis. Many compounds have been hypothesized as primary targets of UV-B action on photosynthesis, including the reaction centre itself, quinone acceptors and redox-active tyrosines [1]. UV-B is also absorbed by the oxygen-evolving Mn cluster and can cause its damage [6].

The reaction to UV depends both on its dose and the irradiation scheme. Acute treatment has a more severe effect than chronic exposure which activates acclimation responses [7, 8]. These responses are aimed at minimizing the impact of UV-B on plant cells. They include leaf thickening, alterations in cuticular wax layers and the biosynthesis of UV-B-absorbing phenolic compounds, such as flavonoids [5]. Leaf yellowing is one of the most visible symptoms of irradiation with high doses of UV-B. It results from chlorophyll degradation and the decreased expression of photosynthesis-related genes. There are similar symptoms during many abiotic and biotic stresses, as well as during natural senescence [9–11]. Many of the stress conditions which cause leaf yellowing also induce the expression of senescence-associated genes (SAGs) [12]. Indeed, UV-B treatment of mature leaves markedly up-regulates the expression of these genes and down-regulates some photosynthesis-related genes [13, 14]. The influence of UV irradiation on plants also depends on their age. DNA damage, measured by homologous recombination events, is clearly more severe in younger *Arabidopsis* [15] and *Nicotiana* plants than in older ones [7]. On the other hand, the decline in anthocyanin, chlorophyll and carotenoid contents as well as in photosynthetic yield is higher in older plants [16, 17].

The effect of UV-B on plant functioning is also affected by environmental conditions (for a review see: [18]). The negative impact of UV irradiation on the growth parameters of cucumber increased with increasing nitrogen fertilization [19]. *Arabidopsis* plants grown in an elevated temperature are more sensitive to UV-B irradiation [20].

There is often a synergistic effect between stresses induced by different factors. Pre-treatment of barley seedlings with other stressors, like a high NaCl concentration minimized the UV-B-induced decrease in the content of photosynthetic pigments and in photosynthetic efficiency [21]. After UV-B pretreatment, plant survival was enhanced under biotic and abiotic stress conditions. Plant tolerance to cold is increased by UV-B as shown by studies both in a growth chamber and in the field [15, 22]. Drought stress also has a lesser impact on plants pre-treated with UV-B [23] (for a review see: [24]). UV-B irradiation can enhance plant resistance to pathogen attack via changing plant morphology, the production of secondary metabolites and the expression of genes controlling pathogen viability [25]. On the other hand, rice plants overexpressing *WRKY89*, a gene induced by pathogen attack, are more resistant to UV-B [26].

The interplay between PAR and UV irradiation is the most widely studied (for a review see: [27]). High light up-regulates the expression of the genes involved in flavonoids synthesis including *PHENYLALANINE AMMONIA-LYASE 1* (*PAL1*) and *CHALCONE SYNTHASE* (*CHS*)*,* as well as the genes encoding ROS scavengers [28]. Flavonoids such as isoflavons and anthocyanins are UV absorbing pigments shown to increase plant tolerance to strong UV irradiation [29]. *Arabidopsis* plants with an impaired production of ascorbate, a ROS scavenger, are more sensitive to UV-B [30]. This suggests that enhanced ascorbate synthesis helps plants to cope with UV-B-induced stress. The resistance of bean plants to elevated UV-B irradiation positively correlates with light intensity [31, 32]. A low dose of UV-B, when supplemented with visible light, does not influence photosynthesis or the expression of photosynthesis-related genes [33]. The chlorophyll content in plants grown in a UV-B-enriched environment may be even 25 % higher than that of the control [34]. The recovery of photosynthesis after UV-B damage is also faster under illumination with photosynthetically active light [35]. UV-B causes the degradation of D1 protein to a 20 kDa fragment which is subsequently completely degraded by proteases in a light-dependent manner. Additionally, *de novo* synthesis of D1 protein occurs only under visible light [35]. Growing plants under visible light supplemented with UV-B activates mechanisms which allow them to survive under subsequent high light stress [36].

Although high doses of UV-B have a negative impact on photosynthetic systems, UV-B is used in post-harvest technology to slow down yellowing during storage [37, 38]. To address this inconsistency we examined the effect of short, 5 min high-dose (8 W·m^−2^) UV-B irradiation on detached *Arabidopsis thaliana* leaves. As light is known to alleviate the effects of UV-B on plants, two different experimental regimes were applied after irradiation. The irradiated samples were kept either in darkness or under constant white light for up to 4 days. To characterize the influence of UV-B on photosynthesis the content of photosynthetic pigments, levels of D1 protein as well as photosynthetic efficiency were analyzed. The production of anthocyanins was examined both at the levels of gene expression and anthocyanin accumulation. Additionally, the expression of the senescence-associated genes, *SAG12*, *SAG13, SENESCENCE1* (*SEN1*) and *WRKY53* was tested. Finally, cell death was checked using trypan blue staining. The results clearly showed that irradiation with a high dose of UV-B can induce two different pathways. The key controlling factor is the presence or absence of visible light after UV-B irradiation.

## Methods

### Plant material

*Arabidopsis thaliana* Columbia-0, and *uvr8-6* (*uvb-resistance 8–6*, SALK_033468, [39]) and *mcp2d-1* (*metacaspase 2d-1*, SAIL_856_D05, [40]) seeds were obtained from The Nottingham Arabidopsis Stock Centre (NASC, Nottingham, UK). Mutant plants were identified by PCR analysis according to standard protocol [41] using Lba1 and gene specific primers listed in Additional file 1: Table S1.

Seeds were sown in Jiffy-7® Peat Pellet (Jiffy International AS, Kristiansand, Norway) and stratified for 2 days at 4 °C. Plants were grown in a growth chamber (Sanyo MLR 350H, Japan) at 23 °C, 80 % relative humidity, with a 10/14 light/dark cycle and fluorescent lamps (FL40SS.W/37, Sanyo) as a light source with a photosynthetic photon flux density of 70 μmol·m^−2^ · s^−1^. Adult leaves from 5–6 week old *Arabidopsis* plants were used for all experiments.

### UV-B treatment

**Fig. 1:**
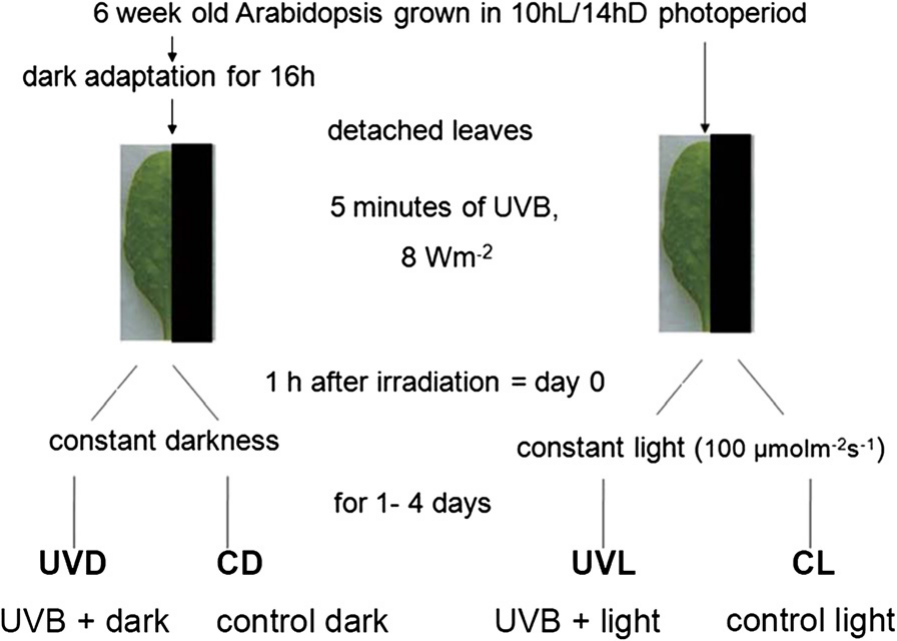
The overall scheme of the experiment. The leaves from 6 week old Arabidopsis thaliana were detached either from dark adapted overnight plants or directly from plants growing in the growth chamber during the light period (2 h after the photoperiodic light had been turn on). The leaves were put on petri dishes on water-soaked paper. Half of each leaf was covered with black paper (control) and the leaves were irradiated with UV-B (8 W·m^−2^) for 5 min. After irradiation the leaves were either left in darkness (leaves from dark-adapted plants) or under continuous illumination with white light (100 μmol ·m^−2^· s^−1^) for up to 4 days. Thus, 4 different kinds of samples were analyzed, i) darkened control (CD), ii) control illuminated with continuous light (CL), iii) UV-B irradiated leaves left in darkness (UVD) and finally, iv) UV-B irradiated leaves kept under continuous illumination (UVL)

Two experimental models were used involving either dark or continuous light treatment. Leaves meant for dark treatment were taken from plants dark-adapted for 16 h prior to the experiment and handled in green safe light. Leaves meant for light treatment were taken directly from the growth chamber during the light period. The irradiation of both kind of samples started at 10 a.m. i.e. 2 h after the photoperiodic light had been turned on. Just before irradiation the *Arabidopsis thaliana* leaves were detached from the plant and put on water-soaked paper. One half of each leaf was covered with black paper (control) and the whole leaf was exposed to 5 min of high intensity UV-B (8 W·m^-^^2^, under USHIO UV-B Lamps G8T5E). After treatment the covers were removed and leaves were transferred either to constant darkness or to constant white light (100 μmol·m^−2^ ·s^−1^) delivered by LEDs (Tops 10 Power Pure White Led OSW4XAHAE1E). After the specified time period leaves were cut into halves and the control and treated halves from 4 different leaves were pooled together, immediately frozen in liquid nitrogen, weighed and kept at −80 °C until further analysis. Each measurement was repeated at least 3 times. Day 0 refers to samples collected 1 h after UV-B treatment. The scheme of the experiment is summarized in Fig. 1.

### Chlorophyll Fluorescence Measurements

Chlorophyll fluorescence in the leaves was imaged using an Open FluorCam FC 800-O/1010 imaging fluorometer (Photon Systems Instruments). Before measurements the leaves were dark-adapted for at least 30 min. The basal fluorescence (F0) was recorded for 5 s, followed by a 1 s pulse of saturating white light (2000 μmol·m^−2^·s^−1^). Data points represent the means of at least 12 leaf halves in 3 independent replicates.

### Pigment extraction

Frozen leaf material was ground in a mortar with 0,5 ml methanol on ice, the extract was collected and the mortar and pestle were washed with an additional 1 ml of methanol. The extract was centrifuged with a table-top centrifuge at 14 000 g for 1 min. The supernatant was transferred to a new tube and the pellet was re-extracted with 0,5 ml methanol twice. All supernatants were combined together and used for the HPLC analysis of photosynthetic pigments. Pellets were extracted on ice with 1 ml of 0,1 % HCl in methanol, centrifuged and re-extracted twice with 0,5 ml of acidic methanol. The supernatants were combined together and their absorption spectra were measured. Anthocyanin content was inferred from absorbance at 532 nm.

### HPLC measurement

HPLC analysis of pigments was done by a method modified from [42].100 μl of methanol pigment extract was loaded with a loop onto a C-18 column (Bionacom Velocity, 5uicrons, 4.6x250 mm), connected to an Akta Purifier (GE Healthcare). The column was pre-equilibrated with 5 ml of solvent A (90 % acetonitrile, 10 % water), and elution was done with following gradient with solvent B (100 % ethyl acetate):

1. 1–5 ml, 100 % A to 80 % A
2. 5–20 ml, 80 % A to 50 % A
3. 20–25 ml, 50 % A to 30 % A
4. 25–30 ml, 30 % A (isocratic)
5. 40–45 ml, 30 % A to 100 % A.

The flow rate was 1 ml/min. Elution was monitored spectrophotometrically at three wavelengths simultaneously (405 nm, 436 nm and 280 nm). Pigments were identified by retention time, compared to standards. The chromatogram analysis and peak integration were done using Unicorn software (GE Healthcare).

For a qualitative determination of pigments, extinction coefficients in HPLC (Additional file 2: Table S2) solvents were determined as follows. Fractions corresponding to pigments of interest were collected separately in a known volume. After recording the spectra in the HPLC solvent, the fractions were dried and resuspended respectively in 80 % acetone—chlorophyll a, chlorophyll b [43], methanol—violaxanthin, lutein [44], ethanol—neoxanthin [45] and hexane—β-carotene [46].

The statistical significance of the differences between treatments was assessed with one-way ANOVA, using GraphPad InStat Software (Additional file 3: Table S3).

### RNA isolation and real-time PCR

RNA isolation, cDNA synthesis and real-time RT-PCR reactions were performed as given elsewhere [47]. All reactions were run in triplicates. The sequence of the primers and their annealing temperatures are listed in Additional file 1: Table S1. A single dark-adapted overnight control sample from day 0 was used as the reference for calculating relative expression levels. The normalization was performed with normalization factors based on the reference gene levels calculated by geNorm v3.4 [48].

### Protein extraction and Western Blot

The leaf material was ground in liquid nitrogen. An extraction buffer (4 % SDS, 2 % β-mercaptoethanol, 2 mM PMSF, 100 mM TrisHCl, pH 8,8) was added in the proportion of 10 μl of extraction buffer per 1 mg of powder mass. The samples were vortexed vigorously, incubated at 80 °C for 3 min, centrifuged for 10 min at 16 000 g at 4 °C and supernatant was mixed with an SDS-PAGE loading buffer. The SDS-PAGE was performed according to [49] in a gel containing 12 % polyacrylamide using the Mini Protean system (Bio-Rad). After separation the proteins were either stained with Coomassie Brilliant Blue staining (for total protein visualization) or transferred to a PVDF membrane (ImmobilonP, Millipore) by the semi-dry transfer method (Trans-Blot SD Semi-Dry Transfer Cell, Bio-Rad) for Western Blot analysis. Membranes were stained with Ponceau S to ensure proper transfer, blocked with 5 % fat free dried milk in PBS with 0,5 % Tween and incubated with an anti-D1 antibody (AS05 084, Agrisera) diluted 1:10 000 for 1 h at room temperature, followed by secondary antibody incubation (Goat anti-rabbit IgG HRP conjugated, Agrisera) under the same conditions. After that a chemiluminescence substrate was added (Clarity Western ECL Substrate, Bio-Rad) and the chemiluminescence was imaged using the BioSpectrum imaging system (UVP).

### Trypan Blue staining

The samples were pretreated (i.e. kept in darkness or left for 2 h under photoperiodic light in the growth chamber), irradiated and kept in either darkness or constant light as described in the “UV-B treatment” section. The only exception was that prior to the irradiation, instead of leaf halves, a middle, narrow part of the detached leaf (perpendicular to the vasculature) was covered with black paper. After the specified time the leaves were covered with 2,5 mg/ml Trypan Blue in lactophenol, heated in a boiling water bath for 1 min, stained at room temperature for an additional 2 h, and destained with a saturated chloral hydrate solution.

## Results

### Effect of UV-B on dark-induced yellowing of Arabidopsis leaves

**Fig. 2:**
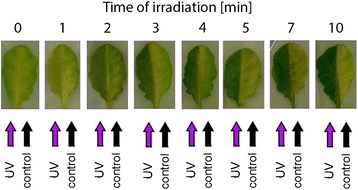
Photographs of the detached leaves of 6-week old *A. thaliana* with one half covered with black paper, and another half irradiated with UV-B (8 W·m^−2^) for the indicated time and left in darkness for 4 days

Different UV-B doses were applied in order to check whether UV-B irradiation can slow down the onset of dark-induced senescence in darkened *Arabidopsis* leaves. Whereas in the non-irradiated leaf halves visible symptoms of senescence, i.e. yellowing, were easy to observe (Fig. 2), UV-B treatment clearly influenced chlorophyll degradation in a dose-dependent manner. Differences in leaf color between the irradiated and non-irradiated halves started to be visible after 3 min of the UV-B (8 W·m^−2^) treatment and persisted up to the 10th minute of irradiation. Based on the results of this preliminary experiment, we decided on a 5 min treatment for further analysis.

**Fig. 3:**
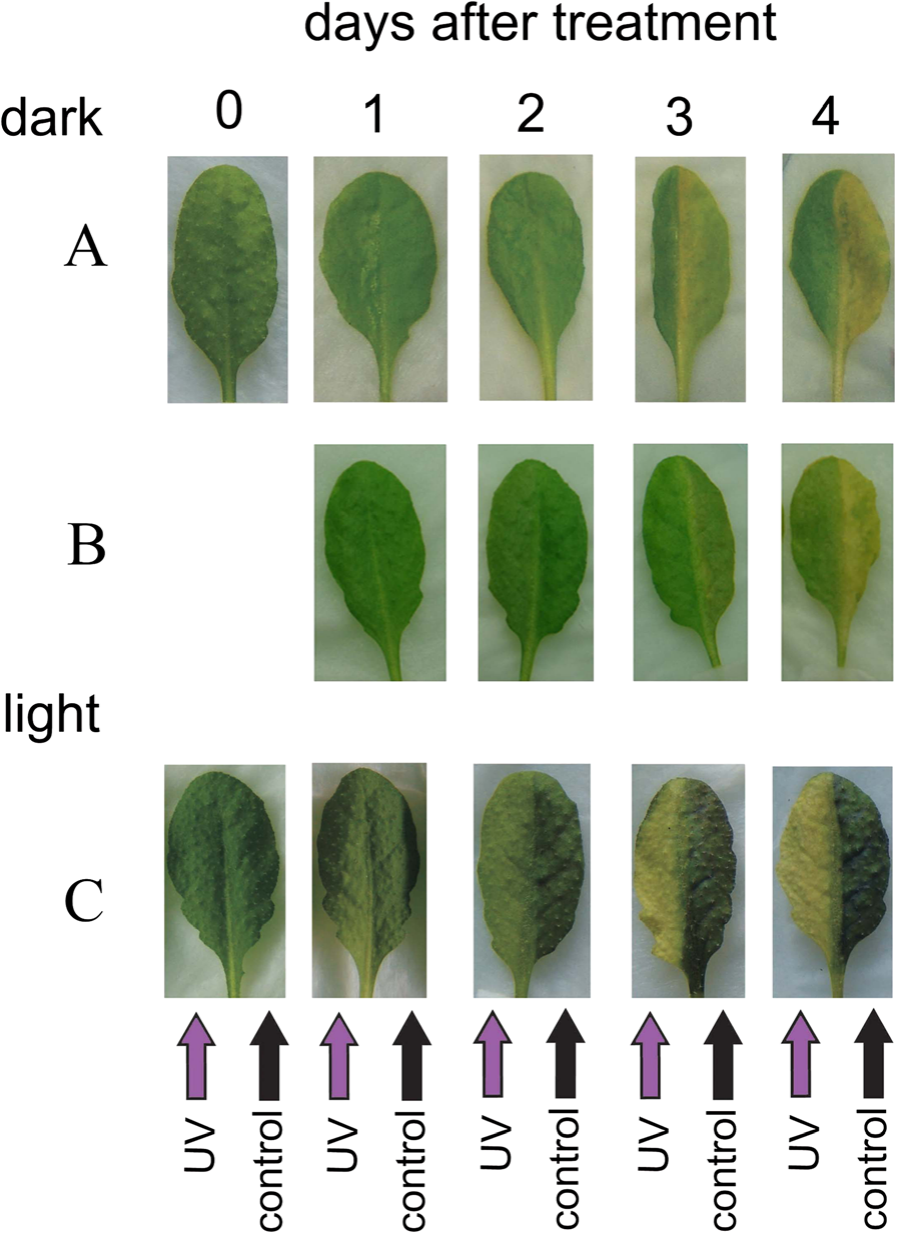
Photographs of detached *A. thaliana* leaves with one half covered with black paper, and another half irradiated with UV-B (8 W·m^−2^) for 5 min and left in darkness (**a** and **b**) or under constant illumination (100 μmol·m^−2^ ·s^−1^ of white light, **c**) for the indicated time. The leaves were taken from plants dark-adapted overnight (**a**), or from plants kept in a growth chamber for 2 h after the dawn (**b** and **c**)

The core idea of the study was to compare the effects of UV-B in dark and light conditions and that was kept in mind when setting up experimental treatments. On the one hand, we wanted to avoid possible effects of the circadian clock. On the other hand, we wanted to test the influence of UVB on either the dark- or light-adapted state of the leaves. Therefore, we decided to start both light and dark experiments at the same time point i.e. 2 h after dawn. In consequence, the plants used for testing the dark-adapted state were kept in darkness for that time. To make sure that this extended night did not result in drastic changes in the observed phenomena, leaf yellowing was observed in leaves taken from plants which were either dark-adapted or kept in photoperiodic light for 2 h before UV-B irradiation, and transferred to darkness afterwards. In both cases chlorophyll degradation was lower in UV-B irradiated leaf halves (Fig. 3, compare a and b), with differences observed only in the rate of degradation which was more prominent in control leaf halves from dark-adapted plants. Thus, for further experiments on the UV-inhibition of dark-induced chlorophyll degradation only dark-adapted plants were used (see below).

### Macroscopic appearance of leaves under different post-treatment light conditions

Two different experimental models were used (Fig. 1). The first of these involved detached leaves from dark-adapted plants. The leaves were UV-B irradiated and kept in darkness for up to 4 days. In the other model leaves were taken from plants 2 h after the start of the light period. They were UV-B irradiated and placed in constant light (100 μmol·m^−2^ ·s^−1^). Dark-induced leaf yellowing was observed in control leaf halves, while those from constant light stayed green but showed reddening, probably due to anthocyanin accumulation (Fig. 3). The opposite effect of post-UV-treatment light conditions was observed in leaf halves irradiated with 8 W·m^−2^ of UV-B for 5 min. In irradiated leaf halves kept in darkness dark-induced chlorophyll degradation was alleviated and yellowing was barely visible even after 4 days. In contrast, leaf halves subjected to UV-B treatment and then transferred to continuous light showed yellowing without the appearance of red coloring. To examine the observed effect in detail different parameters including chlorophyll fluorescence, the expression of senescence-induced and photosythesis-related genes as well as the level of photosynthetic pigments and anthocyanins were investigated.

### Photosynthetic efficiency and photosynthetic pigment content

**Fig. 4:**
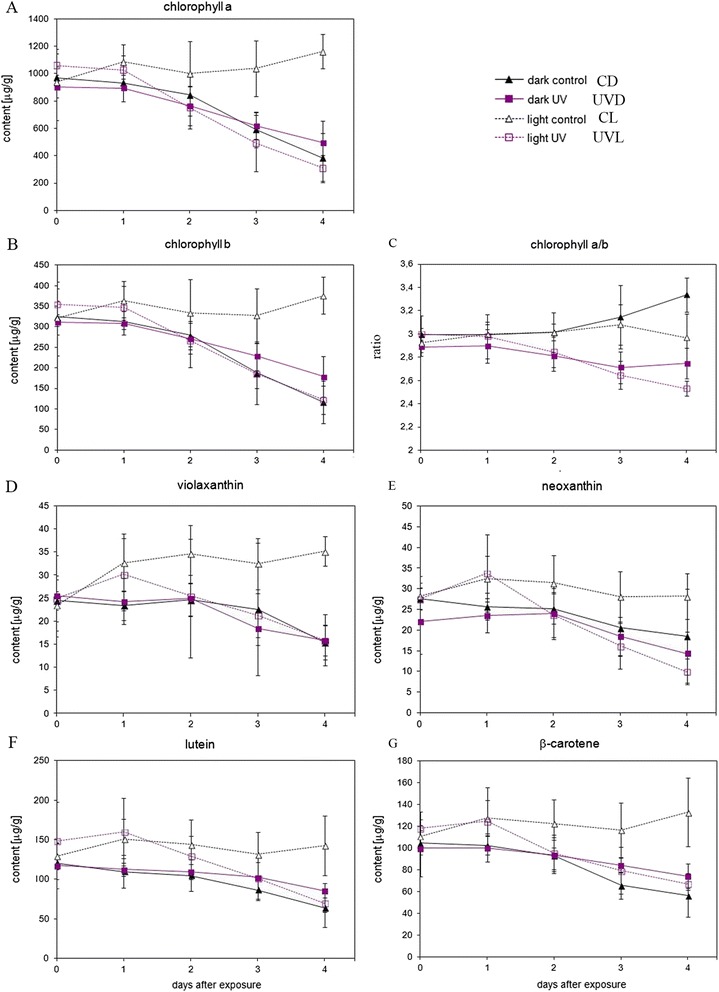
Changes in photosynthetic pigments (**a** chlorohyll a, **b** chlorophyll b, **c** chlorophyll a/b, **d** violaxanthin, **e** neoxanthin, **f** lutein, **g** β-carotene ) in detached *Arabidopsi*s leaf halves either irradiated with UV-B (8 W·m^−2^) for 5 min or covered with black paper (control) and left either in darkness or under constant white light (100 μmol·m^−2^·s^−1^) for the indicated time. Day 0 means 1 h after the treatment. Non-irradiated leaf halves served as a control. Pigments were separated by HPLC with detection by absorbance at 436 nm (chlorophylls) or 405 nm (carotenoids) and their content was determined from the area under the peak of the chromatogram using the extinction coefficients listed in Additional file 2: Table S2. Statistical significance of the differences between treatments was assessed with one-way ANOVA and the results of this analysis are listed in Additional file 3: Table S3

To analyze the changes in pigment composition of the leaves, HPLC analysis of isolated photosynthetic pigments starting from day 0 to day 4 after UV-B treatment was carried out. The results are shown in Fig. 4a and b. The overall changes in the levels of chlorophyll a (chl a) and chlorophyll b (chl b) were similar. In continuous light, starting from the second day, the chlorophyll levels began to drop in the UV-B followed by continuous light (UVL) samples, resulting in a statistically relevant difference between 0UVL and 4UVL, as well as between 1UVL and 4UVL (Additional file 3: Table S3). Meanwhile, in control leaves (control continuous light—CL) the chlorophyll content remained stable or even slightly increased, resulting in a statistically significant difference of *p* < 0.005 between 4UVL and 4CL for both chl a and chl b. The content of both chlorophylls in dark-adapted samples decreased both in treated (UV-B, then darkness—UVD) and un-treated (control darkness—CD) ones, leading to a statistically significant difference of *p* < 0.005 for 4CD vs 4CL and 4UVD vs 4CL for chl a and chl b, as well as lower but still statistically significant differences for the preceding days. However, the dynamic of these processes was different. While in UV-B treated leaves (UVD) the decrease was slow and steady from day 1 on, in the control (CD) it was pretty rapid after 3 days. UV-B treatment slowed down chlorophyll degradation. On day 4 in UVD samples chl a and chl b amounted to 129 % and 153 % of that observed in CD respectively. This difference was not statistically significant. However, whereas a difference between day 0 and day 4 was statistically significant for CD, no statistical significance was observed for UVD. 4 days after irradiation the levels of both chlorophylls were clearly lower in UVL than in CL and similar to CD leaves. During treatment, the chl a/b ratio did not change significantly for CL samples, but increased in CD samples. In both UV-B treated samples this ratio was lower than in the corresponding controls (Fig. 4c). Differences of *p* < 0.005 were noted between day 4 in UVL leaves and its CL control, as well as between 4 UVD and 4CD. Statistically significant differences were found already on 3rd day (i.e. 3UVL vs 3CL, and 3CD vs 3UVD).

Similar trends were observed for all carotenoids tested (Fig. 4d-g). Again, in control samples kept in continuous light, the contents of violaxanthin, lutein and β-carotene increased or stayed unchanged. On the other hand, dark treatment led to a decrease in all carotenoids tested, what manifests as a statistically significant difference between 4CD and 1 to 4 CL. UV-B irradiation either did not influence the effect of darkness (violaxanthin, Fig. 4d) or slightly inhibited it (see: neoxanthin, lutein and β-carotene Fig. 4e-g), although the difference was not statistically significant. After a transient increase on day 1, the decrease in carotenoid levels in UVL leaves on day 4th was either similar (lutein and violaxanthin), 50 % lower (neoxanthin), or slightly higher (β -carotene) than in the darkened control.

**Fig. 5:**
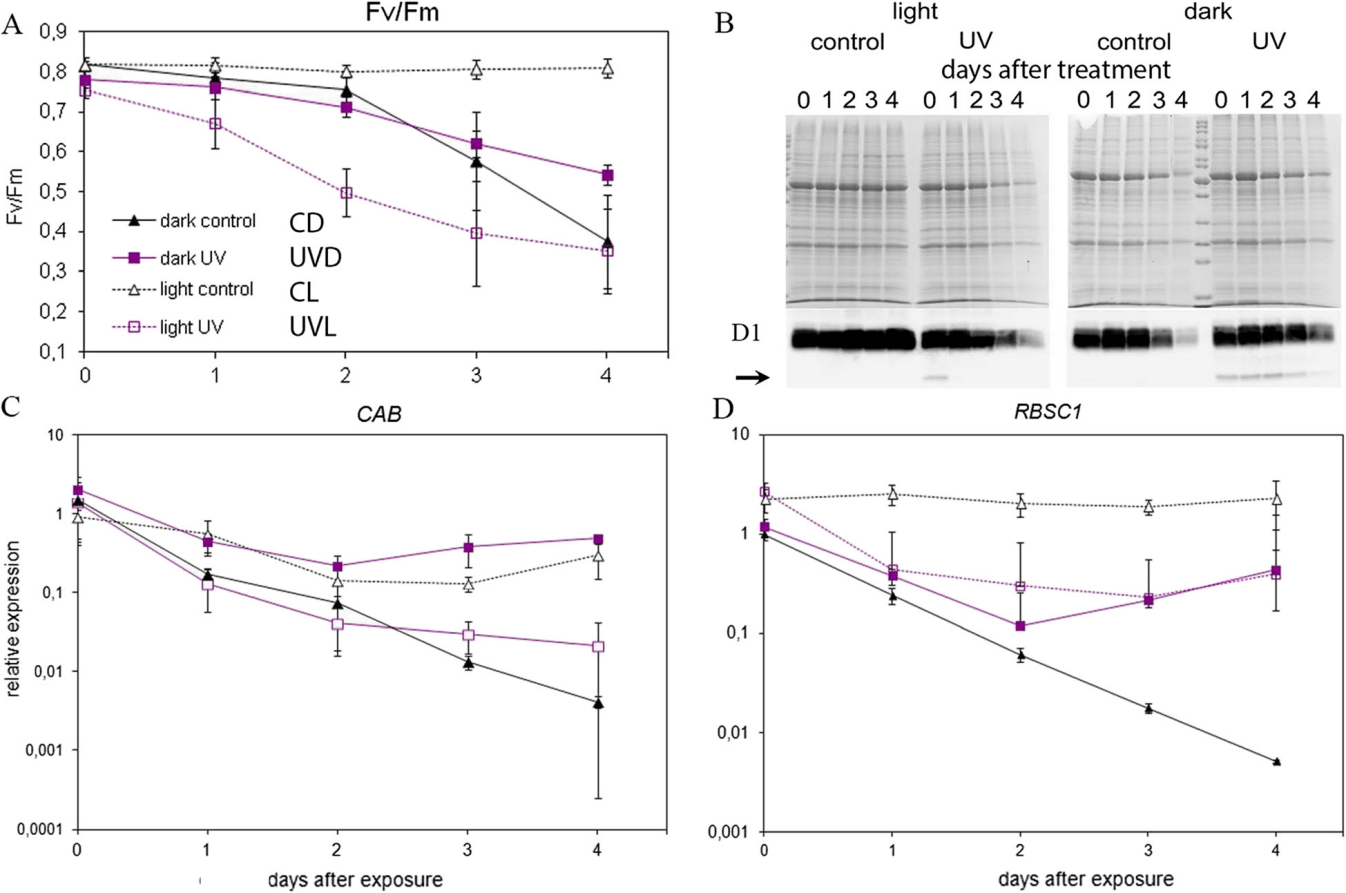
Influence of 5 min UV-B (8 W·m^−2^) irradiation on photosynthesis in *Arabidopsis* leaves. After irradiation samples were kept either in darkness or under constant light (100 μmol·m^−2^·s^−1^) for the given time. Day 0 means 1 h after the treatment. Non-irradiated leaf halves served as a control. **a** Changes in PSII maximal quantum yield (Fv/Fm) during experimental treatment, measured with an imaging fluorometer. The results are the means of measurements for at least 12 different leaves. Statistical significance of the differences between treatments was assessed with one-way ANOVA and the results of this analysis are listed in Additional file 3: Table S3. **b** Total proteins (upper- Coomassie stained SDS-PAGE) and D1 protein (lower- Western blot with anti-D1 antibodies) in examined leaves. Each well contains proteins extracted from 120 mg of tissue The degradation product of D1 protein is marked with an arrow. **c** and **d** Relative expression levels of photosynthesis-related genes (*CAB, RBSC1*) measured with real-time RT-PCR and normalized for the expression of four housekeeping genes (*PDF2, UBC9, UBQ10, SAND*). After the specified time period leaves were cut into halves and control and treated halves from 4 different leaves were pooled. Each measurement was repeated at least 3 times. A single dark-adapted overnight control sample from day 0 was used as a reference for calculating relative expression levels. Error bars indicate the standard error

Bearing in mind the fact that the experimental treatment applied led to a decrease in the photosynthetic pigment content, we examined how these changes influenced photosynthetic performance. We assessed the yield of PSII via the measurement of chlorophyll fluorescence (Fig. 5a). The differences between maximum quantum yield of PSII (QYmax) levels were more clearly visible than these between levels of photosynthetic pigments. QYmax stayed unchanged in the control leaves kept in continuous light (no statistically significant differences between subsequent days in CL leaves). Leaves treated with UV-B prior to being transferred to continuous light showed a fast and very pronounced decrease in QYmax, consistent with the yellowing of the samples. These differences manifest as statistically significant between 3UVL and other leaves from this series (0UVL, 1UVL, 2UVL). In the CD leaves, the quantum yield decreased, first slowly, and from day 3 on, quite rapidly. Leaves treated with UV-B and darkened showed a steady decrease in QYmax, which resulted in higher values of this parameter on day 3 and 4 than in CD leaves (statistically significant difference with *p* < 0.005), which corresponds to the slightly higher amounts of chlorophylls in those samples.

The changes in pigment contents were also accompanied by changes in protein levels. Quantitatively extracted total proteins were separated by SDS-page (Fig. 5b). The amount of proteins decreased in all but CL leaves. The loss of proteins in darkened samples was slower when they were UV-B pre-treated. The amount of D1 protein of PSII was also examined and showed similar trends to total proteins (Fig. 5b). Interestingly, a lower mass product resulting from UV-B-induced degradation could be observed in UV-B-treated samples. This product, present 1 h after irradiation (day 0), was no longer visible after 1 day in the light exposed sample. In darkness its degradation was very slow and the product was still clearly visible even after 4 days.

In order to see if the influence on photosynthetic processes was also reflected at the level of expression of photosynthesis-related genes, quantitative real-time PCR analysis was carried out (Fig. 5c and d). Typical, photosynthesis-related gene transcripts, *RIBULOSE BISPHOSPHATE CARBOXYLASE SMALL CHAIN 1A* (*RBCS1A*) and *CHLOROPHYLL A/B BINDING PROTEINS* (*CABs,* including *CAB1* and *CAB2*), were analyzed (Fig. 5c and d). Whereas the amount of *RBCS1* mRNA stayed unchanged in the CL leaves even after 4 days, it decreased constantly in the darkened control leaves. On day 4 the transcript level of this gene was similar in UVD and UVL samples reaching a level almost 6 times lower than that observed in CL samples. The dark-induced decrease in control leaves was very rapid. After darkness exceeding 4 days (4 days plus overnight pre-treatment) the amount of *RBCS1* reached only 0,3 % of that observed in the leaves kept in continuous light.

The time-course of changes in the *CAB* transcript level was slightly different (Fig. 5c). The steady-state level of this gene decreased during the experiment, with the most drastic drop in the darkened control samples. 4 days after treatment the amount of *CAB* was similar in CL and UVD leaves. The decrease in UVL leaves was clearly faster, reaching only 0,13 % of the transcript present on day 0. Finally, in darkened control leaves, at the end of the experiment, the *CAB* transcript level was only 1,1 % of that present in leaves kept in continuous light.

### Senescence and cell death

**Fig. 6:**
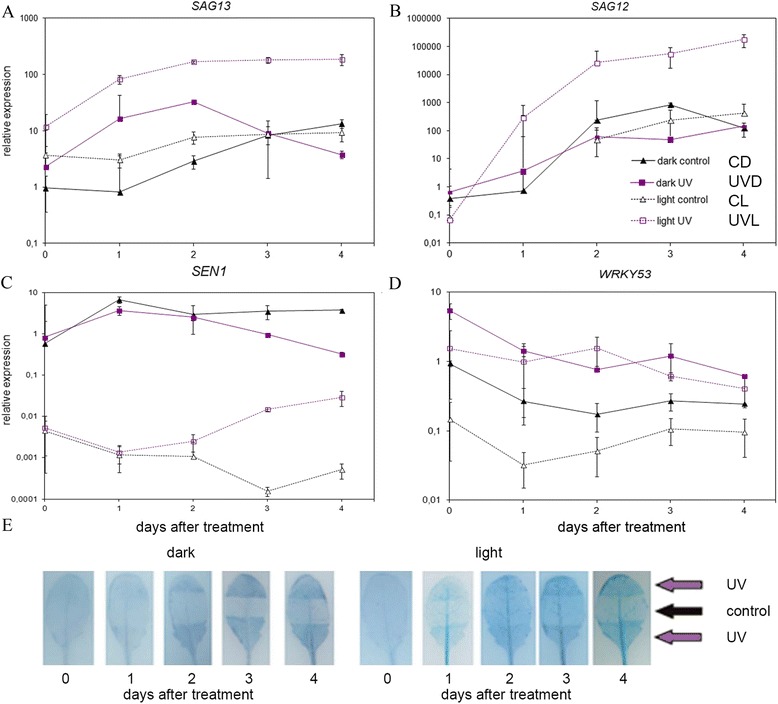
Influence of 5 min UV-B (8 W·m^−2^) irradiation on the senescence and cell death of *Arabidopsis* leaves. After irradiation samples were kept either in darkness or under constant light (100 μmol·m^−2^·s^−1^) for given time. **a**-**d** Time-course of the relative expression of senescence associated genes: (**a**) *SAG13*, (**b**) *SAG12*, (**c**) *SEN1* and (**d**) *WRKY53* normalized for the expression of four housekeeping genes (*PDF2, UBC9, UBQ10, SAND*). Day 0 means 1 h after the treatment. Non-irradiated leaf halves served as a control. After the specified time period leaves were cut into halves and control and treated halves from 4 different leaves were pooled. Each measurement was repeated at least 3 times. A single dark-adapted overnight control sample from day 0 was used as a reference for calculating relative expression levels. Error bars indicate the standard error. **e** Trypan blue staining for cell death of leaves irradiated with UV-B with the middle part covered and transferred either to light or to dark conditions

As leaf yellowing and changes in photosynthetic efficiency often accompany senescence the level of senescence-associated genes (*SAGs*) was also analyzed (Fig. 6). The first of these was *SAG13*, an early senescence marker [12]. Interestingly, only 1 h after the treatment (day 0) the level of this gene was slightly higher in irradiated samples as compared to control ones (Fig. 6a, compare UVD vs CD and UVL vs CL on day 0). The amounts of *SAG13* transcripts increased very strongly in UVL leaves up to the second day, and stayed at the same elevated level on day 3 and 4. Finally, its level was almost 20 times higher than in CL leaves. As in CL, in darkened samples the amount of *SAG13* started to increase between the 1st and 2nd day, but in CD leaves it continued to increase until day 4. An interesting situation was observed for UV irradiated and darkened samples. After strong up-regulation on days 1 and 2, the amount of *SAG13* started to decline, reaching the level similar to CL samples on the 4th day. Nevertheless, this level was still higher as compared to non-irradiated darkened leaves.

The second gene tested was *SAG12*, a late senescence marker [12] (Fig. 6b). The steady-state level of *SAG12* transcript increased strongly in all samples tested in a time-dependent manner. Similarly to *SAG13* the highest level of this gene transcript was observed in UV-B irradiated samples kept in continuous light. After 4 days the amount of this transcript increased by 442 times as compared with CL leaves. Interestingly, the changes in the *SAG12* gene in all but UVL samples were similar, though with slightly different kinetics.

The expression of *SEN1*, another senescence marker was tested in addition to *SAG12* and *SAG13* [50]. Its expression depended mostly on light (Fig. 6c). It was very strongly induced in darkened samples starting from day 0 and lower in samples illuminated with continuous light. Prolonged night caused a very strong up-regulation of *SEN1*, by 130 times as compared to leaves taken directly from the photoperiod 2 h after the light onset (day 0, compare CD and CL). Finally, on the 4th day the amount of *SEN1* transcript was over 7.300 times higher in darkened leaves than in those kept in continuous light. The UV effect started to be visible between the 2nd and 3rd day after irradiation. At this time, the amount of *SEN1* started to decrease in UVD leaves and to increase in UVL ones. 4 days after irradiation the level of this gene transcript was over 11 times higher in CD leaves than in UVD ones. The opposite effect was observed in samples from continuous light. In this case, the level of *SEN1* transcript was 57 times higher in UVL leaves as compared to non-irradiated ones.

The steady-state level of *WRKY53*, a transcription factor up-regulated during early senescence, was also examined. Both darkness and UV-B treatment caused an increase in the level of this gene as compared to samples from constant light (Fig. 6d). Prolonged night caused over a 6-fold increase in the transcript level of this gene (compare CD and CL at the day 0). UV-B acted stronger than darkening, and the effect of UV-B and darkness was synergistic as the strongest, by over 39 times, up-regulation was observed in UVD samples. The amount of *WRKY53* changed over time, decreasing in all but the CL leaves. In control samples from constant light it transiently decreased 1 day after irradiation, but finally reached the same level as on day 0. On the 4th day the highest level of *WRKY53* was observed in both UV-B irradiated samples (6 times higher than in CL ones).

Finally, the occurrence of cell death in the leaves was studied using trypan blue staining (Fig. 6e). UV-B caused the gradual appearance of cell death irrespective of light conditions. Dark-treated leaf parts did not show trypan blue staining until day 4 and even then it was faint compared to that induced by UV-B.

### Anthocyanin content

**Fig. 7:**
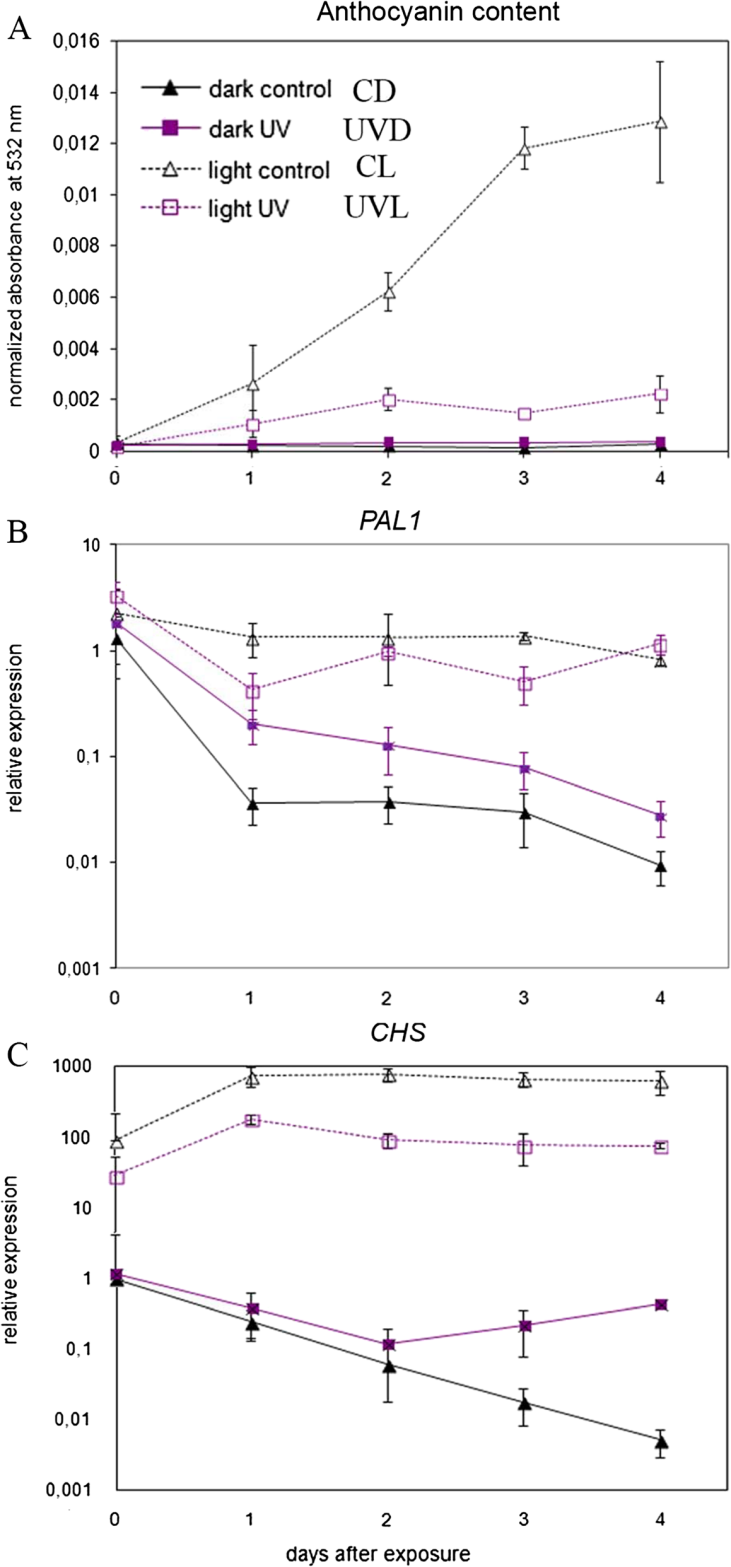
Influence of UV-B on anthocyanins in *Arabidopsis* leaves. After the specified time period leaves were cut into halves and control and treated halves from 4 different leaves were pooled. Each measurement was repeated at least 3 times. Error bars indicate the standard error. **a** Anthocyanin content was analyzed by measuring absorbance at 532 nm and normalized to fresh weight in examined leaves. **b** and **c** Time-course of the relative expression of genes involved in anthocyanin biosynthesis: (**b**) *PAL1* and (**c**) *CHS* normalized for the expression of four housekeeping genes (*PDF2, UBC9, UBQ10, SAND*). A single dark-adapted overnight control sample from day 0 was used as a reference for calculating relative expression levels

It is well known that anthocyanin synthesis is strongly up-regulated not only by visible light but also by UV-B. However, macroscopic observation of the samples treated under our experimental conditions did not confirm this up-regulation. Thus, we checked both the expression of genes involved in anthocyanin synthesis and the content of those pigments more carefully (Fig. 7a). Consistent with visual observations, a very strong increase in the levels of anthocyanins was observed in leaves transferred to continuous light. At the end of experiment, the anthocyanin content in leaves from constant light was 36 times higher than that observed in dark-treated leaves. Interestingly, treatment with UV-B almost completely abolished this response. The anthocyanin level was stable in darkened leaves independent of UV-B irradiation.

We also checked the expression of the genes involved in anthocyanin synthesis, *PAL1* and *CHS*. The expression of *PAL1* was very strongly down-regulated in darkened samples, whereas it stayed nearly unchanged in those undergoing constant illumination (Fig. 7b; CD vs CL). On the 4th day, the level of transcript was almost 200 times higher in leaves from constant light than in those from dark conditions. Interestingly, the effect of darkness was weaker in UV-B irradiated samples. Starting from the 1st day after irradiation the amount of PAL1 transcript was from 1.5 to over 5 times higher in UV-B treated samples than in dark controls.

A similar strong effect of darkness was observed for *CHS* (Fig. 7c). Prolonged night caused a decrease in the mRNA of this gene (day 0). Its level was 90 times higher in illuminated samples (CL) than in darkened ones independent of UV-B treatment (compare CD and UVD). The decrease in *CHS* level in control leaves left in darkness progressed during the experiment. On the 4th day this level was 123 800 times higher in control leaves from constant light than in darkened ones. UV-B down-regulated the *CHS* level in leaves from the light (3–8 times as compared to CL). In dark-treated leaves UV-B up-regulated the amount of this transcript starting from the 2nd day after irradiation.

### Macroscopic appearance of leaves of selected mutants

In order to elucidate possible mechanism(s) underlying the observed UV-B effect, two mutants were examined: *uvr8-6*, depleted of UV-B receptor [39] and *mcp2d*, lacking metacaspase 2d involved in programmed cell death [40].

The former one was used to check the involvement of UVR8-activated signalling pathway in either inhibition or promotion of chlorophyll degradation in darkness and in light respectively. The *mcp2d* mutant served to test the possible role of this metacaspase in chlorophyll degradation in *Arabidopsis* leaves illuminated after irradiation.

The dark-induced leaf yellowing was slowed down in *mcp2d* leaves as compared with WT ones (Additional file 4: Figure S1, dark control). Leaves of *uvr8* plants were more sensitive to UV-B-induced damage. The damage symptoms were more severe in *uvr8* leaves darkened after irradiation. The influence of UV-B on chlorophyll degradation was comparable in WT Col and *uvr8* and *mcp2d* mutants independent on the light conditions (Additional file 4: Figure S1).

## Discussion

### Treatment with high doses of UV-B alleviates darkening-induced senescence symptoms in detached Arabidopsis leaves

One of the most evident symptoms of senescence is yellowing which originates from faster catabolism of chlorophylls in comparison to yellow pigments. This process may be a result of natural senescence or stress (biotic or abiotic). Senescence is also induced in leaves that are detached and stored in darkness or individually darkened on a plant kept in a photoperiod [51]. Yellowing may be delayed by interference with the chlorophyll degradation pathway, as observed in numerous mutants, collectively known as “stay-green”. Those mutants can be classified as either functional, i.e. those with delayed overall senescence, or cosmetic, i.e. those where only chlorophyll degradation is delayed while other aspects of senescence progress normally [52].

In our model, the darkening of detached leaf halves resulted in a decrease in both chl a and chl b content and an increase in the chl a/chl b ratio. These trends are similar to those observed before [5, 53]. However, we did not observe such a significant amount of chlorophyll degradation products as previously reported [53]. Some amount of pheophytins was found only after 4 days of darkness (not shown).

A much higher retention of carotenoids is typical of the senescing leaves of almost all plants. Usually, the carotenoid/chlorophyll ratio increases [54]. Changes in the levels of neoxanthin, violaxanthin, lutein and β-carotene occur in parallel [55, 56]. Our results are consistent with those observations. The level of carotenoids did not change during the first two days of darkening, and started to decrease from the 3rd day. Taken together, these results indicate that our system is an appropriate experimental model of senescence.

Sub-lethal doses of UV-C, UV-B and gamma irradiation are widely used in post-harvest technology. Beneficial, hormetic effects of such irradiation include the sanitization of fresh vegetables and fruits, an increase in phenolic compounds, in the content of lycopene and other pigments, the up-regulation of antioxidant content and antioxidant enzyme activity (for a rewiev see: [57]). Treatment with ultraviolet light may also influence chlorophyll content. Irradiation with a relatively high dose of UV-B (8.8 kJ ﻿·m^-^2) has been shown to delay the yellowing of broccoli florets ([37]) and lime peel during storage [38]. This yellowing results from the storage of detached plant parts in darkness. It has been shown that dark storage induces senescence and the expression of senescence-associated genes in broccoli starting after 3 days of storage [58].

We show that a similar dose-dependent effect can be observed in detached *Arabidopsis* leaves kept in darkness after irradiation with 8 W·m^−2^ UV-B (i.e. 2,4 kJ·m^−2^) (Fig. 2). Our results indicate that high-doses of UV-B interfere with chlorophyll degradation in darkness and slow it down. The contents of all photosynthetic pigments tested, except neoxanthin and violaxanthin, were slightly lower in irradiated leaves during the first 2 days after treatment (Fig. 4). However, starting from day 3 chlorophyll degradation in control leaves progressed much faster and the balance reversed in favor of UV-B treated plants. The same was observed for photosynthesis efficiency (Fig. 5a). The maximal quantum yield of PSII was higher in UV treated leaves than in control (only darkened) ones. Thus, starting from 3 days after irradiation the positive effects of the treatment outweighed the negative ones. Chlorophyll a and b were degraded in darkened samples but the degradation rate of both pigments was modulated by UV irradiation. The chl a/chl b ratio increased in darkened control leaves (Fig. 4b), in accordance with previous reports [53]. This probably results from the faster degradation of LHCII-derived (Light-harvesting complex II) chlorophylls mediated by a complex of STAY-GREEN1 (SGR1) with chlorophyll catabolic enzymes [59], as it is counteracted in a mutant with impaired SGR1 protein function (*nonyellowing1-1, nye1-1*) during mild stress treatment [60]. Interestingly, in UV-treated samples the chl a/chl b ratio remained almost unchanged during the whole time-course of the experiment. This suggests that in the absence of visible light UV-B activated signals can interfere with a specific pathway of LHCII degradation. In our system the Lhcb1 protein level remained unchanged during the experiment (Western blot data not shown). It has been observed that the degradation of Lhc proteins starts later than degradation of D1 protein [60]. It is likely that the components of LHCII, namely proteins and pigments, are degraded sequentially and that chlorophyll degradation precedes other processes.

The amounts of β-carotene and lutein were slightly higher after UV irradiation (Fig. 4f and g). These pigments are constituents of the photosynthetic protein pigment complexes, PSII and LHCII respectively. The increased retention of β-carotene after UV-B treatment correlates with the slower degradation of D1 protein and may result from the increased stability of the whole complex.

Lutein and other xanthophylls which build LHCII complexes are important in photoprotection mechanisms [61, 62]. The higher level of lutein and unchanged level of violaxanthin after UV-B pretreatment may result from a slower degradation of LHCII. Neoxanthin was the only pigment that showed an immediate decrease after UV-B treatment. It is known that neoxanthin absorbs UV and that it can photoisomerize upon excitation [63]. Although other carotenoids also can photoisomerize, the effect for neoxanthin is the strongest and can reach 10 % of this pigment’s content. Thus, the observed decrease may result from photoisomerization. Light is necessary for an effective conversion of neoxanthin isomers to the primary conformation. Since there is no light in our system, the process is very slow and the level of neoxanthin in UV treated samples remains lowest during the whole time-course of the experiment.

All UV effects on photosynthesis in darkened leaves were accompanied with a slower decrease in total protein level, a slower decrease in the amount of D1 protein as well as a slower decrease in the level of photosynthesis-related transcripts (Fig. 5). This is consistent with observed changes in photosynthetic efficiency. The specific product of UV-induced D1 protein cleavage was present after the treatment (Fig. 5b, day 0). As in [35], we showed the persistence of this product in the absence of light, in our case for up to 4 days. Taken together, our results suggest that UV-B treatment alleviates the effects of darkening on the functionality of the photosynthetic apparatus.

The examination of expression levels of senescence-associated genes showed that the UV effect observed was not caused by a simple retardation of senescence (Fig. 6). On the one hand, the mRNA levels of S*AG12* and *SEN1* were lower after short UV-B treatment in samples darkened for 3 days, when the differences in chlorophyll degradation started to be visible. On the other hand, over the whole experiment the expression of *WRKY53* was elevated in irradiated samples. The amount of *SAG13* transcripts was higher for the first 2 days after the treatment and started to decrease on 3rd day. This rather complicated pattern of senescence-associated gene regulation suggests that UV-B interferes with their expression; it does not supplement darkening-induced senescence but tends to modulate it.

It should be noted that the advantageous effects of UV started to be visible at the same time as cell death symptoms appeared, confirmed by trypan blue staining. This shows that in our experimental system leaves are highly susceptible to both the damaging effects of UV-B and to the beneficial ones. The exact mechanism of the inhibition of dark-induced leaf yellowing by high doses of UV remains to be determined. To date, studies on the UV-dependent inhibition of chlorophyll degradation have been performed using broccoli florets and lime peel [37, 38]. It has been shown that UV-B inhibits the activity of chlorophyll peroxidase [37] and recently, the specifically affected by UV-B chlorophyll peroxidase C has been identified [64].

Our results demonstrated that the inhibition of chlorophyll degradation in darkness was independent of the UV-B photoreceptor, UVR8, since leaves of the *uvr8* mutant showed the same symptoms as those of WT plants i.e. slowed dark-induced chlorophyll degradation (Additional file 4: Figure S1, dark). The more severe damage observed in *uvr8* mutants probably results from the lack of UVR8-regulated protective mechanisms It is in line with the hypothesis that UVR8 mediates responses to low UV-B doses, whereas high doses of UV-B activate an independent pathway involving the mitogen-activated protein kinase (MAPK) cascade [3]. This cascade regulates UV-B dependent programmed cell death (PCD) in plants. One of the regulators of cell death induced during biotic and abiotic stresses in *Arabidopsis* is metacaspase 2d (MCP2d). Experiments employing the *mcp2d* mutant demonstrated that the effects of UV-B observed in this mutant were comparable to the wild type (Additional file 4: Figure S1, dark). Thus, the inhibition of dark-induced chlorophyll degradation was not due to the inhibition of the activity of this metacaspase.

### Visible light influences UV-B action

Visible light dramatically affected the response of leaves to high doses of UV. While darkened leaves stayed green even 4 days after UV irradiation, yellowing was observed in leaves transferred to continuous light starting from the 2nd day (Fig. 3). This yellowing was a result of a decrease in the levels of all photosynthetic pigments and was accompanied by a decrease in photosynthesis efficiency (Figs. 3 and 5). In contrast to the senescing CD samples, in UVL ones the chl a/chl b ratio decreased. The decrease resembled the effect observed in the *nye1-1* mutant during mild salt stress (Sakuraba et al. 2014). This suggests that the level of activation of different pigment degradation pathways during UV-mediated senescence in light was not the same as during dark-induced senescence. In particular, the specific, SGR1-dependent pathway of LHCII degradation appeared not to be activated.

The cumulative dose of UV-B necessary to decrease the chlorophyll level has been calculated for *Pisum sativum* as 300 kJ·m^−2^ [65]. In *Arabidopsis* grown in a 12 h light photoperiod with photosynthetic photon flux density (PPFD) of 300 μmol·m^−2^ ·s^−1^ plus 6 kJ·m^−2^·d^−1^ of UV-B [34], the chlorophyll content increased [34]. Even the addition 2,4 W·m^−2^ of UV-B for 5 h (i.e. 8,64 kJ·m^−2^ dose) to 40 μmol·m^−2^ ·s^−1^ of PPFD did not influence the chlorophyll level in 29 day old *Arabidopsis* [66]. In this experiment the content of both chlorophylls, lutein, violaxanthin and antheraxanthin remained unchanged as long as 4 days after UV-B irradiation. The UV-B dose used in our experiment e.g. 2,4 kJ·m^−2^ was comparable with above experiments. The main differences were: using detached leaves from plants grown in a shorter (10 h of light) photoperiod and using constant illumination after the treatment. However, when leaves after UV-B treatment were transferred back to the photoperiod, the same symptoms were observed, but with slower kinetics (data not shown). The influence of PAR intensity during *Arabidopsis* growth on the effect of UV-B has been shown before [67, 68]. The light intensity during the growth was lower in our experiment (70 μmol·m^−2^ ·s^−1^) than those reported by [66] (130 μmol·m^−2^ ·s^−1^) and [34] (300 μmol·m^−2^·s^−1^). Thus, it is possible that either light intensity and/or the duration of the photoperiod modulate the UV-B effects on photosynthesis (compare [28]). Additionally, Götz et al. [68] showed that a low level of UV-B during growth leads to a low accumulation of protective isoflavonoids. This may lead to a higher susceptibility of the plants to UV-B irradiation, compared to the plants grown in the presence of higher UV-B levels. While in our growth chamber UV-B irradiation was completely excluded, no data on the intensity of UV-B during plant growth are provided either by Moon’s or Poulson’s groups [34, 66].

The expression of senescence-associated genes has been shown to be up-regulated in *Arabidopsis* grown under white light supplemented with UV-B [13]. Similarly, in our system the expression of all senescence-associated genes tested was strongly up-regulated in UVL leaves (Fig. 6). A comparison of the results obtained for darkened and illuminated samples shows that PAR is a key factor in the induction of senescence-associated genes by UV-B. In consequence, the observed decrease in photosynthesis resulting from chlorophyll degradation seems to be a result of the initiation of the senescence process.

Interestingly, UV-B pretreatment completely inhibited the accumulation of anthocyanins in continuous light. The expression of anthocyanin regulatory genes as well as anthocyanin accumulation have been shown to be strongly increased by UV-A and by visible light, mainly in the blue range. UV-B alone is much less effective, but it acts synergistically with visible light [29, 69, 70]. In our experiments the expression levels of *PAL1* and *CHS* were elevated in leaves continuously illuminated with white light as compared to darkened samples (Fig. 7). UV-B either did not influence (*CHS*) or slightly reduced (*PAL1*) this increase. In contrast, while anthocyanin accumulation was very strongly enhanced by white light, UV-B pretreatment counteracted this effect. The activation of the senescence program by UV-B irradiation, might eliminate the need for the production of photoprotective pigments.

Although the impact of UV-B on photosynthesis, chlorophyll degradation, expression of senescence-associated genes was modulated by the subsequent light conditions, cell death was observed in samples both darkened and illuminated with continuous light. Visible light proved to be necessary for the proper course of a senescence program triggered by a high dose of UV-B. Light is required for the onset of cell death under nutrient-limiting conditions [71] and in *Arabidopsis* protoplasts after UV-C treatment [72]. Chloroplast delivered signals, most probably ones connected with the production of reactive oxygen species, seem to be involved in executing senescence and cell death [73–75]. Indeed, in our system the senescence program leading to cell death was initiated in illuminated samples. In darkened ones other processes leading to cell death were activated. The signaling pathways leading to cell death after UV-B irradiation and darkening do not act synergistically but seem to be mutually exclusive to some extent.

Again, UVR8-dependent signalling pathway was not involved in UV-B-induced chlorophyll degradation in light (Additional file 4: Figure S1).

## Conclusions

Our results show the importance of the light conditions applied after the irradiation with high dose of UV-B. These conditions influenced the expression of photosynthesis-related and senescence-associated genes, chlorophyll degradation and photosynthesic efficiency. Short UV-B treatment promoted leaf yellowing in light and inhibited it upon the storage of leaves in darkness. However, irrespective of light conditions, visible cell death symptoms appeared 3 days after UV-B irradiation.

## Additional files

Additional file 1: Table S1. Sequences of primers used in this study. (DOCX 15 kb) Additional file 2: Table S2. Extinction coefficients in HPLC solvent. (DOCX 10 kb) Additional file 3: Table S3. Analysis of statistical relevance in changes of photosynthetic pigments level (a: chlorophyll a, b: chlorophyll b, c: chlorophyll a/chlorophyll b ratio, d: violaxanthin, e: neoxanthin, f: lutein, g: β-carotene) as well as in yield of Photosystem II (h). (PDF 325 kb) Additional file 4: Figure S1. Photographs of the detached leaves of 6-week old *A. thaliana* WT, *mcp2d* and *uvr8* mutants with one half covered with black paper, and another half irradiated with UV-B (8 W · m^−2^) for 5 min and (A) left in darkness or (B) illuminated with white light (100·μmol·m^−2^·s^−1^) for 4 days. (TIF 10951 kb)

## Abbreviations

*CAB*: *CHLOROPHYLL A/B BINDING PROTEIN*
CD: Control dark, non-treated leaves dark adapted and left in the darkness
chl: Chlorophyll
*CHS*: *CHALCONE SYNTHASE*
CL: Control light, non-treated leaves left under continuous light (100 μmol·m^−2^_ ·_^s^^−1^)
LHC II: Light-harvesting complex II
*mcp2d*: *metacaspase 2d*
*nye1-1*: *non-yellowing1-1*
*PAL1*: *PHENYLALANINE AMMONIA-LYASE 1*
PAR: Photosynthetically active radiation
PPFD: Photosynthetic photon flux density
PSII: Photosystem II
QYmax: Maximum quantum yield of Photosystem II
*RBCS1A*: *RIBULOSE BISPHOSPHATE CARBOXYLASE SMALL CHAIN 1A*
ROS: Reactive oxygen species
*SAG*: *SENESCENCE ASSOCIATED GENE*
*SEN1*: *SENESCENCE1*
SGR1: STAY-GREEN1
UV: Ultraviolet
UVD: UV dark—dark adapted leaves irradiated for 5 min with 8 W·m^−2^ of UV-B and left in darkness
UVL: UV light—leaves irradiated for 5 min with 8 W·m^−2^ of UV-B and left under continuous light (100 μmol·m^−2^ ·s^−1^)
*uvr8*: *uvb-resistance 8*
