# Supplementary material for: The effect of UV-B on Arabidopsis leaves depends on light conditions after treatment
Source: BMC Plant Biol. 2015 Nov 25;15:281. doi: 10.1186/s12870-015-0667-2 (PMC4660668; doi:10.1186/s12870-015-0667-2)
Supplement: Additional file 1: Table S1. — Sequences of primers used in this study. (DOCX 15 kb) [file 12870_2015_667_MOESM1_ESM.docx]

| Name | sequence 5'-3' | annealing  T [°C] | reference |
| --- | --- | --- | --- |
| uvr8-6 LP | TTTGCTTGAACCATCCGTTAG | 50 |  |
| uvr8-6 RP | AATGGCATTGACTTCAGATGG |  |  |
| mcp2d-1 LP | AAGCATTCCCATTAATCCACC | 50 |  |
| mcp2d-1 RP | AATGTCTCGTTGAACGGTACG |  |  |
| Lba1 | TGGTTCACGTAGTGGGCCATCG | 50 |  |
| RBCS1AF | TTCGGAATCGGTAAGGTCAGG | 63 | [73] |
| RBCS1AR | AACGGCGGAAGAGTTAACTGC |  |  |
| CAB_RTF | CCAGAGGCATTCGCTGAGTTG | 53 | [73] |
| CAB_RTR | CCTTACCAGTGACGATGGCTTG |  |  |
| SAG12RT2F | GTGTCTACGCGGATGTGAAG | 53 | [74] |
| SAG12RT2R | CAGCAAACTGATTTACCGCA |  |  |
| SAG13_RTF | CTCTTCTCGTGACCAACGAGTG | 53 |  |
| SAG13_RTR | GCTTGAATATTGACGTTCCCAC |  |  |
| SEN1_RTF | CACCTCTACAAACATGTGGATC | 53 | [73] |
| SEN1_RTR | GTTGTCGTTGCTTTCCTCCATC |  |  |
| WRKY53_RTF | CAGACGGGGATGCTACGG | 53 |  |
| WRKY53_RTR | GGCGAGGCTAATGGTGGTG |  |  |
| UBQ10F | GGCCTTGTATAATCCCTGATGAATAAG | 53 | [75] |
| UBQ10R | AAAGAGATAACAGGAACGGAAACATAGT |  |  |
| UBCF | CTGCGACTCAGGGAATCTTCTAA | 53 | [75] |
| UBCR | TTGTGCCATTGAATTGAACCC |  |  |
| PDF2F | TAACGTGGCCAAAATGATGC | 53 | [75] |
| PDF2R | GTTCTCCACAACCGCTTGGT |  |  |
| SANDF | AACTCTATGCAGCATTTGATCCACT | 53 | [75] |
| SANDR | TGATTGCATATCTTTATCGCCATC |  |  |

The *CAB* primers anneal with the sequences of two genes belonging to the family of chlorophyll a/b binding proteins, i.e Cab1 (Lhcb1.3 At1g29930) and Cab2 (Lhcb1.1, At1g29920).
