# Supplementary material for: The effect of UV-B on Arabidopsis leaves depends on light conditions after treatment
Source: BMC Plant Biol. 2015 Nov 25;15:281. doi: 10.1186/s12870-015-0667-2 (PMC4660668; doi:10.1186/s12870-015-0667-2)
Supplement: Additional file 2: Table S2. — Extinction coefficients in HPLC solvent. (DOCX 10 kb) [file 12870_2015_667_MOESM2_ESM.docx]

| pigment | λ [nm] | Extinction coefficient [Lg^-1^cm^-1^] |
| --- | --- | --- |
| chlorophyll a | 436 | 85,8 |
| chlorophyll b | 436 | 65,75 |
| β-carotene | 405 | 51,6 |
| lutein | 405 | 83,5 |
| neoxanthin | 405 | 111,3 |
| violaxanthin | 405 | 108,1 |
