# Supplementary material for: The effect of UV-B on Arabidopsis leaves depends on light conditions after treatment
Source: BMC Plant Biol. 2015 Nov 25;15:281. doi: 10.1186/s12870-015-0667-2 (PMC4660668; doi:10.1186/s12870-015-0667-2)
Supplement: Additional file 3: Table S3. — Analysis of statistical relevance in changes of photosynthetic pigments level (a: chlorophyll a, b: chlorophyll b, c: chlorophyll a/chlorophyll b ratio, d: violaxanthin, e: neoxanthin, f: lutein, g: β-carotene) as well as in yield of Photosystem II (h). (PDF 325 kb) [file 12870_2015_667_MOESM3_ESM.pdf]

Supplemental Table 3. Analysis of statistical relevance in changes of photosynthetic pigments level (a: chlorophyll a, b: chlorophyll b, c: chlorophyll a/chlorophyll b ratio, d: violaxanthin, e: neoxanthin, f: lutein, g: β-carotene) as well as in yield of Photosystem II (h).

a

Chl a

	0CL	1CL	2CL	3CL	4CL	0CD	1CD	2CD	3CD	4CD	0UVD	1UVD	2UVD	3UVD	4UVD	0UVL	1UVL	2UVL	3UVL	4UVL
**0CL**										2										3
**1CL**									1	3				1	2				2	3
**2CL**										2					1				1	3
**3CL**										3					2				2	3
**4CL**									2	3				2	3				3	3
**0CD**										2					1				1	3
**1CD**										2										2
**2CD**										1										2
**3CD**		1			2											1				
**4CD**	2	3	2	3	3	2	2	1				1				3	3			
**0UVD**																				
**1UVD**										1										2
**2UVD**																				2
**3UVD**		1			2															
**4UVD**		2	1	2	3	1										2	1			
**0UVL**									1	3					2				2	3
**1UVL**										3					1				2	3
**2UVL**																				
**3UVL**		2	1	2	3	1										2	2			
**4UVL**	3	3	3	3	3	3	2	2				2	2			3	3			

One-way ANOVA with Tukey post-test
empty cell- not significant
1: P<0.05
2: P<0.01
3: P<0.001

b

Chl b

	0CL	1CL	2CL	3CL	4CL	0CD	1CD	2CD	3CD	4CD	0UVD	1UVD	2UVD	3UVD	4UVD	0UVL	1UVL	2UVL	3UVL	4UVL
**0CL**										2										2
**1CL**									1	3					2				1	3
**2CL**										3										2
**3CL**										2										2
**4CL**									2	3					2				2	3
**0CD**										2										2
**1CD**										2										2
**2CD**										1										
**3CD**		1			2											1	1			
**4CD**	2	3	3	2	3	2	2	1				2				3	3			
**0UVD**																				2
**1UVD**										2										2
**2UVD**																				
**3UVD**																				
**4UVD**		2			2											1	1			
**0UVL**									1	3					1				1	3
**1UVL**									1	3					1				1	3
**2UVL**																				
**3UVL**		1			2											1	1			
**4UVL**	2	3	2	2	3	2	2									3	3			

One-way ANOVA with Tukey post-test

empty cell- not significant

1: $P<0.05$

2: $P<0.01$

3: $P<0.001$

c

Chl a/Chl b

	0CL	1CL	2CL	3CL	4CL	0CD	1CD	2CD	3CD	4CD	0UVD	1UVD	2UVD	3UVD	4UVD	0UVL	1UVL	2UVL	3UVL	4UVL
**0CL**																				
**1CL**																				1
**2CL**																				1
**3CL**															1				2	3
**4CL**																			1	2
**0CD**																				
**1CD**																				1
**2CD**																				1
**3CD**														1					2	3
**4CD**											1	1	2	3	3			2	3	3
**0UVD**										1										
**1UVD**										1										
**2UVD**										2										
**3UVD**									1	3										
**4UVD**				1						3										
**0UVL**																				1
**1UVL**																				1
**2UVL**										2										
**3UVL**				2	1				2	3										
**4UVL**		1	1	3	2		1	1	3	3						1	1			

One-way ANOVA with Tukey post-test
empty cell- not significant
1: P<0.05
2: P<0.01
3: P<0.001

d

Violaxanthin

	0CL	1CL	2CL	3CL	4CL	0CD	1CD	2CD	3CD	4CD	0UVD	1UVD	2UVD	3UVD	4UVD	0UVL	1UVL	2UVL	3UVL	4UVL
**0CL**																				
**1CL**										1					1					
**2CL**										2					1					1
**3CL**										1					1					1
**4CL**										2				1	2					2
**0CD**																				
**1CD**																				
**2CD**																				
**3CD**																				
**4CD**		1	2	1	2															
**0UVD**																				
**1UVD**																				
**2UVD**																				
**3UVD**					1															
**4UVD**		1	1	1	2															
**0UVL**																				
**1UVL**																				
**2UVL**																				
**3UVL**																				
**4UVL**			1	1	2															

One-way ANOVA with Tukey post-test
empty cell- not significant
1: P<0.05
2: P<0.01
3: P<0.001

e

Neoxanthin

	0CL	1CL	2CL	3CL	4CL	0CD	1CD	2CD	3CD	4CD	0UVD	1UVD	2UVD	3UVD	4UVD	0UVL	1UVL	2UVL	3UVL	4UVL
**0CL**															2					
**1CL**															2				1	1
**2CL**																				3
**3CL**																				2
**4CL**																				2
**0CD**																				2
**1CD**																				1
**2CD**																				1
**3CD**																				
**4CD**																	1			
**0UVD**																				
**1UVD**																				
**2UVD**																				
**3UVD**																				
**4UVD**	2	2															2			
**0UVL**																				1
**1UVL**										1					2				1	3
**2UVL**																				
**3UVL**		1															1			
**4UVL**		1	3	2	2											1	3			

One-way ANOVA with Tukey post-test
empty cell- not significant
1: P<0.05
2: P<0.01
3: P<0.001

f

Lutein

	0CL	1CL	2CL	3CL	4CL	0CD	1CD	2CD	3CD	4CD	0UVD	1UVD	2UVD	3UVD	4UVD	0UVL	1UVL	2UVL	3UVL	4UVL
0CL										1										
1CL									1	3					1					2
2CL										2										2
3CL										2										1
4CL										3										2
0CD																				
1CD																				
2CD																				
3CD		1														1				
4CD	1	3	2	2	3											3				3
0UVD																				
1UVD																				
2UVD																				
3UVD																				
4UVD		1														1				2
0UVL									1	3					1					2
1UVL																				3
2UVL																				1
3UVL																				
4UVL		2	2	1	2					3					2	2	3	1		

One-way ANOVA with Tukey post-test

empty cell- not significant

1: P<0.05

2: P<0.01

3: P<0.001

g

β-carotene

	0CL	1CL	2CL	3CL	4CL	0CD	1CD	2CD	3CD	4CD	0UVD	1UVD	2UVD	3UVD	4UVD	0UVL	1UVL	2UVL	3UVL	4UVL
**0CL**																				
**1CL**									2	2					1					2
**2CL**										2										1
**3CL**										2										
**4CL**									1	3			1	2	3			1	2	3
**0CD**																				
**1CD**																				
**2CD**																				
**3CD**		2			1											1	1			
**4CD**		2	2	2	3											2	2			
**0UVD**																				
**1UVD**																				
**2UVD**					1															
**3UVD**					2															
**4UVD**		1			3															
**0UVL**									1	2										
**1UVL**									1	2										1
**2UVL**					1															
**3UVL**					2															
**4UVL**		2	1		3												1			

One-way ANOVA with Tukey post-test

empty cell- not significant

1: P<0.05

2: P<0.01

3: P<0.001

h

QYmax

	0CL	1CL	2CL	3CL	4CL	0CD	1CD	2CD	3CD	4CD	0UVD	1UVD	2UVD	3UVD	4UVD	0UVL	1UVL	2UVL	3UVL	4UVL
0CL									3	3			2	3	3		3	3	3	3
1CL									3	3			2	3	3		3	3	3	3
2CL									3	3				3	3		3	3	3	3
3CL									3	3			1	3	3		3	3	3	3
4CL									3	3			1	3	3		3	3	3	3
0CD									3	3			2	3	3		3	3	3	3
1CD									3	3				3	3		2	3	3	3
2CD									3	3					3			3	3	3
3CD	3	3	3	3	3	3	3	3		3	3	3	3	3		3	1		3	3
4CD	3	3	3	3	3	3	3	3	3		3	3	3			3	3	3		
0UVD									3	3				3			2	3	3	3
1UVD									3	3				3	3		1	3	3	3
2UVD	2	2		1	1	2			3	3				1	3			3	3	3
3UVD	3	3	3	3	3	3	3	3	3		3	3	1			3		3	3	3
4UVD	3	3	3	3	3	3	3	3				3	3			3	3		3	3
0UVL									3	3				3	3			3	3	3
1UVL	3	3	3	3	3	2	2		1	3	2	1			3			3	3	3
2UVL	3	3	3	3	3	3	3	3		3	3	3	3	3		3	3		1	3
3UVL	3	3	3	3	3		3	3	3		3	3	3	3	3	3	3	1		
4UVL	3	3	3	3	3	3	3	3	3		3	3	3	3	3	3	3	3		

One-way ANOVA with Tukey post-test
empty cell- not significant
1: P<0.05
2: P<0.01
3: P<0.001
