# Supplementary figures and images for: The effect of UV-B on Arabidopsis leaves depends on light conditions after treatment
Source: BMC Plant Biol. 2015 Nov 25;15:281. doi: 10.1186/s12870-015-0667-2 (PMC4660668; doi:10.1186/s12870-015-0667-2)

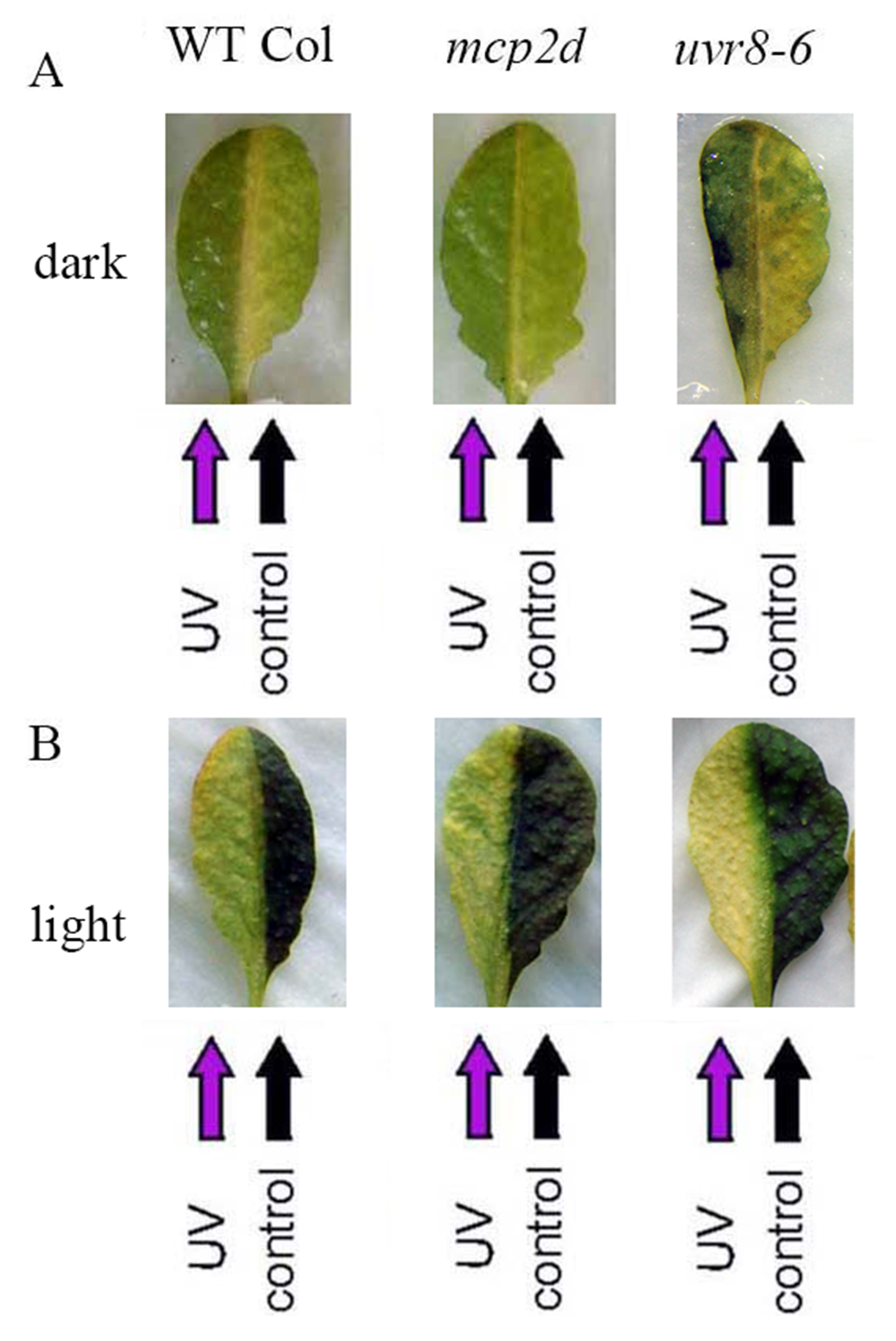

Supplement: Additional file 4: Figure S1. — Photographs of the detached leaves of 6-week old A. thaliana WT, mcp2d and uvr8 mutants with one half covered with black paper, and another half irradiated with UV-B (8 W · m−2) for 5 min and (A) left in darkness or (B) illuminated with white light (100·μmol·m−2·s−1) for 4 days. (TIF 10951 kb) [file 12870_2015_667_MOESM4_ESM.tif]
